# An Integrated Deep Learning and Molecular Dynamics Simulation-Based Screening Pipeline Identifies Inhibitors of a New Cancer Drug Target TIPE2

**DOI:** 10.3389/fphar.2021.772296

**Published:** 2021-11-23

**Authors:** Haiping Zhang, Junxin Li, Konda Mani Saravanan, Hao Wu, Zhichao Wang, Du Wu, Yanjie Wei, Zhen Lu, Youhai H. Chen, Xiaochun Wan, Yi Pan

**Affiliations:** ^1^ Center for High Performance Computing, Joint Engineering Research Center for Health Big Data Intelligent Analysis Technology, Shenzhen Institutes of Advanced Technology, Chinese Academy of Sciences, Shenzhen, China; ^2^ Shenzhen Laboratory of Human Antibody Engineering, Institute of Biomedicine and Biotechnology, Shenzhen Institutes of Advanced Technology, Chinese Academy of Sciences, University City of Shenzhen, Shenzhen, China; ^3^ Center for Cancer Immunology, Shenzhen Institutes of Advanced Technology, Chinese Academy of Sciences, University City of Shenzhen, Shenzhen, China

**Keywords:** TIPE2, UM-164, virtual screening, deep learning, molecular dynamics simulation

## Abstract

The TIPE2 (tumor necrosis factor-alpha-induced protein 8-like 2) protein is a major regulator of cancer and inflammatory diseases. The availability of its sequence and structure, as well as the critical amino acids involved in its ligand binding, provides insights into its function and helps greatly identify novel drug candidates against TIPE2 protein. With the current advances in deep learning and molecular dynamics simulation-based drug screening, large-scale exploration of inhibitory candidates for TIPE2 becomes possible. In this work, we apply deep learning-based methods to perform a preliminary screening against TIPE2 over several commercially available compound datasets. Then, we carried a fine screening by molecular dynamics simulations, followed by metadynamics simulations. Finally, four compounds were selected for experimental validation from 64 candidates obtained from the screening. With surprising accuracy, three compounds out of four can bind to TIPE2. Among them, UM-164 exhibited the strongest binding affinity of 4.97 µM and was able to interfere with the binding of TIPE2 and PIP2 according to competitive bio-layer interferometry (BLI), which indicates that UM-164 is a potential inhibitor against TIPE2 function. The work demonstrates the feasibility of incorporating deep learning and MD simulation in virtual drug screening and provides high potential inhibitors against TIPE2 for drug development.

## Introduction

Tumor necrosis factor (TNF)-alpha-induced protein 8 (TNFAIP8 or TIPE) family of proteins is believed to be regulators of innate and adaptive immunity, as well as cell proliferation, inflammation, and cell death (Lou and Liu, 2011; Goldsmith and Chen, 2017; Yuan et al., 2020). As a member of the TNFAIP8 family, TIPE2 protein is mainly expressed in the placenta and lymphoid tissues (Padmavathi et al., 2018). To maintain immune homeostasis, TIPE2 controls immune cell activation, migration, and apoptosis (Luan et al., 2014). TIPE2 is found to promote Fas-mediated T cell apoptosis (Liu MW. et al., 2015). Depending on the cell types involved, TIPE2 may act as a tumor suppressor (Zhang et al., 2016) or enhancer through, for example, MDSCs (myeloid-derived suppressor cells) (Yan et al., 2017, 2020). Therefore, inhibiting TIPE2 in certain cell types has the potential to treat certain cancers, e.g., lung cancer (Bordoloi et al., 2019). Importantly, TIPE2 is a new target of STAT3, and inhibiting its expression in MDSCs helps enhance T cell activation in tumors (Yan et al., 2017). This indicates that inhibitors of TIPE2 may exert an anti-cancer effect through MDSCs, and hence, it would be medically meaningful to discover TIPE2 inhibitors. We previously identified TIPE2 as a new therapeutic target for cancer immunotherapy since it plays a critical role in the functional polarization of MDSCs (Yan et al., 2020). To the best of our knowledge, despite the significance of TIPE2 in preventing some cancers, no compounds have demonstrated inhibition against TIPE2 function to date.

The high-resolution crystal structure of TIPE2 protein (cytoplasmic protein) was solved in 2009 with an uncharacterized fold that is different from the predicted fold of a DED (Zhang et al., 2009). TIPE2 protein is made up of 184 amino acid residues and is mainly made up of helices. It consists of six antiparallel helices in which helix 5 contains a kink (caused by pro153) and is broken into two short helices (helix 5a and 5b), forming the base of the helical bundle. Surprisingly, TIPE2 contains a huge hydrophobic central cavity, which helps in cofactor binding, and this cavity is found to undergo competitive cofactor binding to activate the immune response to maintain immune dynamic balance. From the crystal structures, it is believed that the unique structural features of TIPE2 protein determine its unique biological functions (Zhang et al., 2010). The availability of a high-resolution experimental structure can aid greatly in identifying novel potential drug candidates against TIPE2 protein.

In general, the drug discovery process against a disease takes a long time and is a costly process. The advances in machine learning methods, especially deep learning, will shorten the drug discovery time, and the researchers in this field will develop more and more applications that solve well-defined problems in the near future. Virtual screening is one field that greatly benefits from the development of deep learning; however, Drug–Target (DTA) prediction is the core component of virtual screening, which directly determines its accuracy and efficiency. To improve the performance of DTA prediction, a model based on deep learning has been developed by the Wei research group (Kaushik et al., 2020). In another interesting study, the authors selected KEAP1 protein, a hot protein in the tumor research field, as a virtual screening target for a database containing 1.3 billion ligands (Gorgulla et al., 2020). However, the ability of the VirtualFlow platform developed in this study lies in its tens of thousands of combined CPUs for molecular docking and cloud computing platforms.

In our very recent work, we built a DL model, “DeepBindRG,” for categorizing native-like protein-ligand complex (Zhang et al., 2019b). We used a complex figure-like 2D input representation and ResNet architecture. We also propose a strategy for virtual screening inhibitors against SARS-CoV-2 3C-like protease (Zhang et al., 2020a). Most importantly, we recently demonstrated that a hybrid method can efficiently be used for drug repurposing for SARS-CoV-2 (Zhang et al., 2020b). It first demonstrates that deep learning-based methods combined with MD simulations are promising strategies to aid the drug development process, especially in identifying novel inhibitors or modulators against therapeutic protein targets.

In our present study, we have built a TIPE2-ligand complex model based on the crystal structure of TIPE2. With the binding pocket from the complex model, we have carried out a large-scale virtual screening against three datasets. Compared to our previous drug repurposing virtual screening against RdRp of SARS-CoV-2, we have a very large data size and have carried much more MD simulations. Since TIPE2 is much smaller than RdRp, it allows us to carry out MD simulation of the full protein instead of the simplified pocket MD simulation to achieve higher accuracy in acceptable computational consumption.

The ability of our hybrid virtual screening pipeline developed in this study lies in its highly efficient protein-ligand binding prediction algorithms and several stages of screening strategies, which emphasizes a gradual shift from efficiency on a large scale to accuracy in the later stage. Finally, the authors considered four out of 69 candidates for experimental validation. They found one compound with a low-micromolar binding affinity with TIPE2, which is an excellent candidate for further optimization, and two compounds with a relatively high-micromolar binding affinity. Among them, UM-164 has a binding affinity of 4.97 µM. Our work has shown the promising future of applying deep learning-based methods and MD simulation-related methods in large-scale drug lead discovery.

Incorporating deep learning and MD simulation helps achieve the balance between accuracy and efficiency in virtual drug screening. Currently, there are very few works that combined large-scale deep learning and MD in drug screening. Our previous drug repurposed against RdRp was the first attempt (Zhang et al., 2020b), but the compound database is relatively too small to sufficiently demonstrate the power of this hybrid method in large-scale drug screening. This time, we have screened over more than 8 thousand compounds and have carried more than 60 standard MD simulations, which is on a much larger scale compared to screening over ~1 thousand approved drugs and only 12 pocket MD simulations in the previous work.

## Materials and Methods

A previously developed deep learning and molecular simulation-based hybrid strategy is used for virtual screening against TIPE2 over several compound datasets. The step-by-step virtual screening pipeline results in 69 high potential binding candidates with TIPE2. Among them, four were considered to validate the efficacy.

### Structural Modeling of TIPE2 and Compound Datasets

The TIPE2 sequence was obtained from UniProt Consortium (2021), and the TIPE2-ligand model was constructed by I-TASSER (Zhang, 2008). Here, we intended to model the holo (ligand-bound) conformation, given that the currently available high-resolution crystal structure of TIPE2 is an apo structure. The ligand was taken from the template protein (PDB ID: 4xk8) (Zhou et al., 2008) by the COFACTOR algorithm (Roy et al., 2012) within the I-TASSER using structure comparison and protein-protein networks, later manually shifting the position to avoid atoms clash. We extract the amino acids within 1 nm of the ligand as the binding pocket. The three TargetMol datasets (Targetmol-Approved_Drug_Library, Targetmol-Natural_Compound_Library, and Targetmol-Bioactive_Compound_Library) and a compiled in-house dataset were used as virtual screening libraries.

### Molecular Vector-Based Drug Screening

**FIGURE 1:**
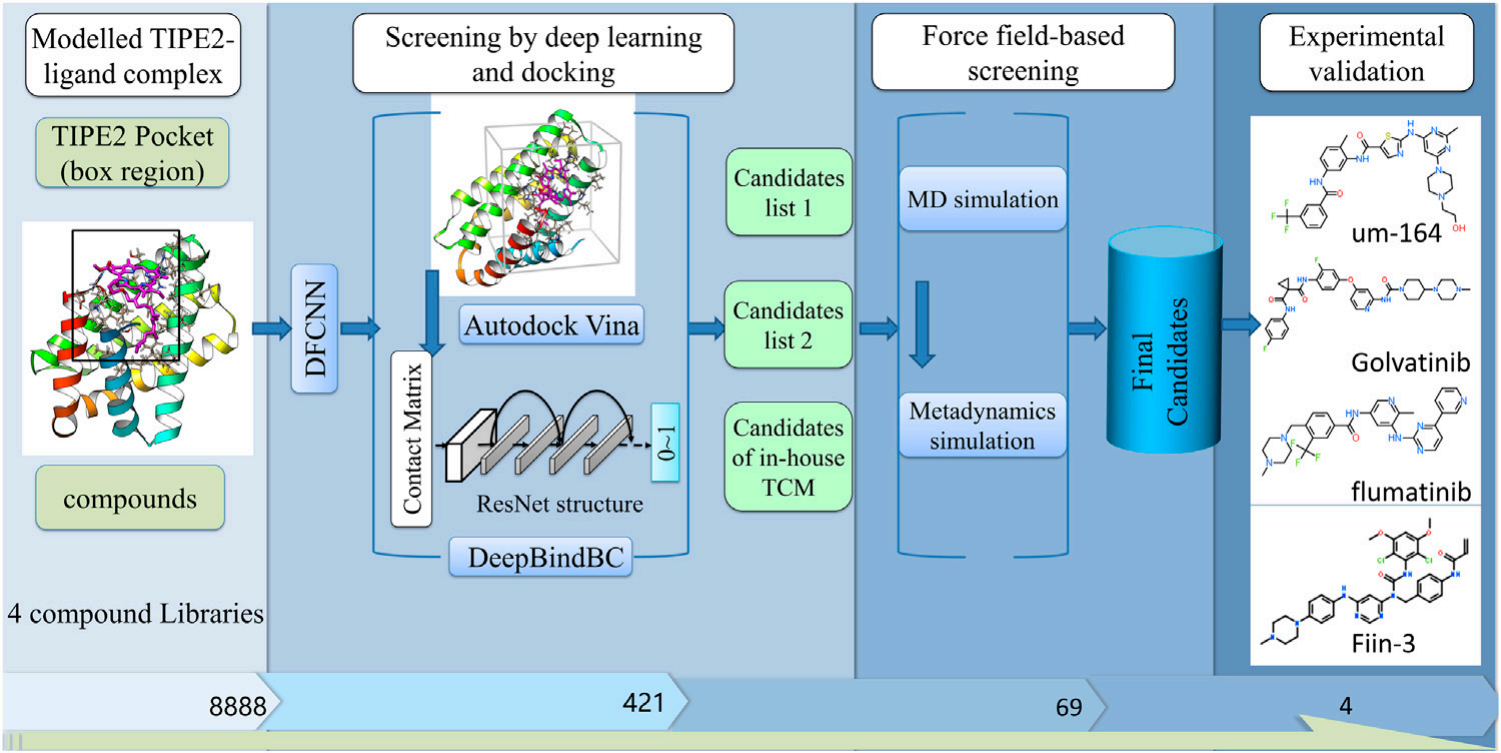
The diagram of our screening pipeline. The screening procedures include preliminary screening by deep learning and docking, fine screening by force field-based methods, and fine experimental validation.

A deep learning-based method, DFCNN (Dense fully Connected Neural Network), has been developed for predicting protein-drug binding probability (Zhang et al., 2019a) and used in this article for the initial drug screening (Figure 1A). DFCNN has used concise mol2vec representation for the protein pocket and ligand, compared to many other methods. It is not dependent on protein and ligand binding conformation, which has greatly accelerated the prediction efficiency. Moreover, it avoids misleading outcomes caused by wrong predicted binding conformation as other methods often encountered. Here, we have used the training set mean and deviation values for the normalization of testing data. The loaded model weights of DFCNN are from the previously completed training. As described in our previous article (Zhang et al., 2019a), the DFCNN model was trained on a dataset extracted from the PDBbind database (2017 version) (Liu Z. et al., 2015). The input representation is the concatenated molecular vector of protein pocket and ligand, and all the molecular vectors are generated by mol2vec (Jaeger et al., 2018), which is inspired by the word2vec model in natural language processing. The positive data are directly from the experimental known protein-ligand pairs, whereas the negative data are created by cross-combination of proteins and ligands from the PDBbind database. After finishing the training, we obtained a model with well-trained weight, which can be used in future applications. We had written homemade scripts to prepare the model input and model application, respectively. A directly loaded trained DFCNN model is enough to run the prediction; no extra parameter settings are needed. The virtual screening procedure by DFCNN is similar to that in our previous work, which is drug repurposing against RdRp of SARS-CoV-2 (Zhang et al., 2020b). DFCNN achieved an AUC value around 0.91 for the independent testing set, which has a much better performance compared to the other four machine learning methods, that is, SVM (AUC of 0.6729), RandomForest (AUC of 0.8444), xgboost (AUC of 0.8601), and CNN (AUC of 0.8642) (Zhang et al., 2019a). The model is about ~100,000 times faster than Autodock Vina (Trott and Olson, 2010) in predicting protein-ligand binding probability (range 0–1), mostly due to no need to explore the protein-drug complex conformation. We have previously validated the efficiency and effectiveness of the DFCNN by missing the known active compounds in a dataset of ~10 million compounds and checking DFCNN’s ability to recall the known active compounds by scoring. The following formula is used:

$$Ratio\_0.9=P_{tpr0.9}/random_{0.9}=\left(N_{0.9}/N_{total}\right)/\left(NN_{0.9}/N\_all\right),$$ (1)

where $N_{0.9}$ is the number of active compounds with scores higher than 0.9; $N_{total}$ is the total number of active compounds for each protein. The prediction TPR ( $\text{P}\_{\text{tpr}}_{0.9}$ ) is defined by $N_{0.9}/N_{total}$ . $NN_{0.9}$ is the total number of compounds with scores above 0.9, N_all is the total number of compounds used in the test. The random guess rate ( $random_{0.9}$ ) is defined as $NN_{0.9}/\text{N}\_\text{all}$ . $P\_tpr_{0.9}/random_{0.9}$ was defined as the prediction-random ratio with the cutoff of 0.9 (Ratio_0.9). Our previous test has chosen a representative target of the DUD.E dataset. The result shows six out of eight protein targets, Ratio_0.9 is greater than 1.4, and the top Ratio_0.9 value is 860, indicating that DFCNN can enrich the active compounds for many kinds of targets in pools of ten million compounds (Zhang et al., 2020b).

### Structure-Based Drug Screening

DeepBindBC, an in-house deep learning-based software, is used for structure-based drug screening. Unlike the DFCNN, the input of DeepBindBC includes both the physical-chemical information and spatial information between the protein-ligand interfaces (Figure 1A); hence, DeepBindBC can achieve higher accuracy but requires protein-drug complex structure information as input generated by Autodock Vina.

Autodock Vina is used to dock the target with the potential ligands (Steffen et al., 2010). The pocket is determined by the location of the ligand in the template protein. Moreover, we set the cavity volume space at 2.5, 2.5, and 2.5 nm in x, y, and z dimensions from the pocket mass center. AutoDock Tools were used to convert the PDB file format to the PDBQT file format (Morris et al., 2009). The exhaustiveness was set to 8; the num_modes was set to 20, and the energy range was set to 3. The scoring function and optimization algorithm of Autodock Vina has been well discussed in a previous article (Steffen et al., 2010).

The DeepBindBC is a ResNet model trained over the PDBbind database. In DeepBindBC, the protein-ligand interaction interface information will be converted into figure-like metrics, similar to DeepBindRG (Zhang et al., 2019b). By incorporating the cross-docking (docking proteins and ligands from different experimental complexes) conformation as negative training data, DeepBindBC is highly possible to distinguish non-binders.

### Force Field-Based Screening

Further drug screening was carried out by force field-based molecular dynamics (MD) simulations. In this study, we selected 69 compound binding complexes, which were predicted candidates by previous deep learning methods, for MD simulation, respectively. Binding free energy calculation can be estimated by metadynamics simulations to explore whether protein-ligand will bind in solution. Metadynamics relies on the addition of a bias potential to sample the free energy landscape along with a specific collective variable of interest (Laio and Gervasio, 2008; Saleh et al., 2017). The detailed MD simulation and followed metadynamics simulation were illustrated in Supplementary Material Section S1.

### Tools Used in Analysis

The USCF Chimera, VMD, ICM-browserPro, and Discovery Studio Visualizer 2019 were used to generate the structure and visualize the 2D protein-ligand interactions (Humphrey et al., 1996; Pettersen et al., 2004; BIOVIA, 2005; icm_browser_pro, 2020).

### Bio-Layer Interferometry

Recombinant TIPE2 proteins were purchased from the Abcam company and biotinylated by the EZ-Link biotinylation reagent (Thermo Fisher Scientific). Briefly, TIPE2 proteins and biotinylation reagent were mixed with a 1:1 molar ratio and then incubated for 2 h at 4°C. The mixture was purified by 3K MWCO dialysis cassettes (Thermo Fisher Scientific) to remove unreacted biotinylation reagent.

Affinities (K_D_ values) were determined by BLI using an OctetK2 instrument (PALL ForteBio). Biotinylated TIPE2 proteins in PBS were loaded onto Super Streptavidin (SSA) biosensors (ForteBio). Average saturation response levels of 5 nm were observed in 10 min for TIPE2 proteins. PBS with 0.1% BSA, 0.01% Tween-20, and 1% DMSO was prepared as the assay buffer. Sensors with TIPE2 proteins were then washed in assay buffer for 10 min to remove nonspecifically bound proteins and establish stable baselines before starting association-dissociation cycles with small molecules. DMSO only references were included in all assays. Raw kinetic data collected were processed in the Data Analysis software provided by the manufacturer using double reference subtraction in which both DMSO only reference and an inactive reference were subtracted. The resulting data were analyzed based on a 1:1 binding model from which kon and koff values were obtained and then K_D_ values were calculated.

### Competitive BLI

PIP2 (phosphatidylinositol 4,5-bisphosphate) was purchased from the Echelon company. To measure competitive binding to TIPE2 protein between PIP2 and UM-164 by BLI, two concentrations (5 and 25 μM) of UM-164 were added to PBS with 0.01% Tween-20 as two sets of assay buffers, respectively, and the interference patterns from the assay buffer without UM-164 and a biosensor without TIPE2 protein were used as two of controls. TIPE2 proteins (10 μg/ml) in three assay buffers were loaded to Streptavidin (SA) biosensors (ForteBio) for 60 s and flowed 150 μM of PIP2 for the 60 s, respectively. The competitive characteristics were analyzed by the Data Analysis software.

## Results

The screening pipeline is illustrated in Figure 1. Here, we first identified the potential ligand binding pocket and then used a deep learning-based method and docking to do a fast preliminary screening. After that, molecular dynamics simulation-based methods were used to further shortlist the results. We have gone over the final candidates and have a preference for those that have a good score and a strong interaction based on a 2D snapshot. Finally, we selected four compounds from the final candidates and carried out experimental validation. However, the other compounds that were not chosen for experiment validation; nevertheless, they have a good chance of binding the TIPE2. The result shows that among the four compounds, one has a low-micromolar binding affinity with a K_D_ value of 4.97 µM, two compounds have a binding affinity with a K_D_ value of 189.1 and 298.3 µM, and one has a weak binding affinity with a K_D_ value of 858,100 µM.

Unlike many other drug screening pipelines, our screening strategy is highly hybrid and takes full usage of different methods’ advantages to achieve a balance between accuracy and efficacy. In the deep learning screening section, we have used the molecular vector-based method DFCNN first since it is highly efficient as it does not rely on docking structure, making it quite suitable for large-scale preliminary drug screening (Zhang et al., 2019a, 2020a, 2020b; Majumdar et al., 2021). The output list from DFCNN was used for docking and structure-based deep learning prediction by the DeepBindBC method (Zhang et al., 2020b), which makes the prediction more robust. In the force field-based screening section, we first carry MD simulations for the predicted protein-ligand complexes from the output of the deep learning screening section. After that, metadynamics simulation was continued from previous results to further examine the binding free energy. The MD simulation results can provide predicted atomic interaction details and binding stability; meanwhile, metadynamics simulation predicted protein-ligand binding free energy landscape. Altogether, this pipeline provides a highly efficient and accurate way to identify inhibitors against TIPE2. The molecular diversity is extremely large; hence, screening over some large databases would be desirable. This pipeline provides important clues that may help to virtual screening over billions of compounds databases while keeping the balance between accuracy and efficacy.

**FIGURE 2:**
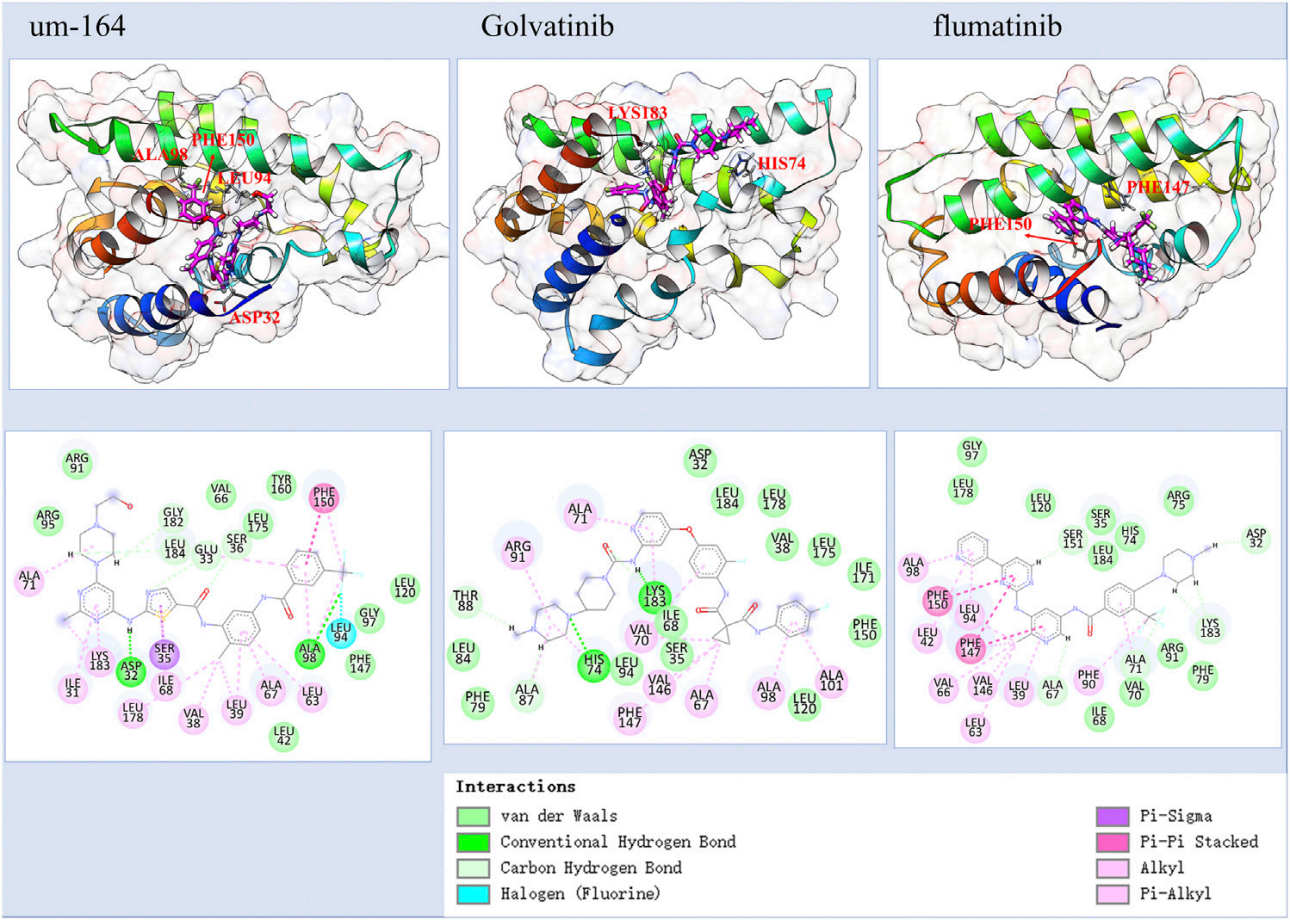
The predicted atomic interaction details of the three experimental validated potent compounds, among them, UM-164 has the strongest binding affinity with a K_D_ value of 4.970 µM.

Figure 2 and Supplementary Figure S1 together display the interaction between four selected compounds that interacted with the TIPE2 from the last frame of 100 ns MD simulation. We observed that the UM-164 has two hydrogen bonds, a Pi–Pi stacking, and a halogen interaction with the TIPE2 and has many other non-hydrophobic interactions. The hydrogen bonds are formed with ASP32 and ALA98. The Pi–Pi interaction was formed with PHE150. This strongly supports the experimental result that UM-164 has the strongest binding affinity among the four test cases, with a K_D_ value of 4.97 × 10^−06^.

Golvtinib was also predicted to have formed hydrogen bond interaction with HIS74 and LYS183, respectively. The binding of flumatinib seems mostly maintained by Pi–Pi interactions and other types of hydrophobic interactions. It forms Pi–Pi interaction with PHE150 and PHE147, respectively. This supports the findings that golvatinib and flumatinib have binding affinities with K_D_ values of 189.1 and 298.3 µM, respectively. Fiin-3 formed one hydrogen bond with ASP32 and formed Pi–Pi interaction with PHE150, as shown in Supplementary Figure S1; however, experimental results show that it only has a very little reactivity against TIPE2.

### Preliminary Screening by Deep Learning-Based Methods and Docking

In a step-by-step manner, we obtain TIPE2 inhibitor candidates with high predicted values from DFCNN and DeepBindBC and with a low docking score. Sixteen compounds were selected with the criteria of DeepBindBC>0.99, Docking<=−10, and DFCNN>0.99, as shown in Table 1. It is found that Sennoside_A has very high DeepBindBC and DFCNN scores. Meanwhile, it also has a very low docking score, which indicates that the compound has a high potential to bind with the TIPE2. According to the prediction scores, most of the other listed compounds all have a very high chance of binding with TIPE2.

**TABLE 1:** The potential inhibitors of TIPE2 from the three TargetMol datasets (DeepBindBC>0.99, Vina Docking<=−10, and DFCNN>0.99). The table was ranked by DeepBindBC score in descending order.

Name	DeepBindBC	Vina docking	DFCNN
Sennoside_A	1	−11.3	0.9908
Sennoside_B	1	−10.4	0.9908
Dabrafenib	0.9996	−10.2	0.994
Olmutinib	0.9994	−10.6	0.9971
CEP37440	0.9992	−10.1	0.9967
GW_4064	0.9991	−11.2	0.9976
Omipalisib	0.9991	−10.6	0.9907
FLT3-IN-1	0.9985	−10.2	0.9968
Gedatolisib	0.9984	−11	0.9967
Avitinib	0.9984	−10.2	0.9955
RO_46-8443	0.9978	−10.3	0.9944
VLX1570	0.9972	−10.5	0.993
Orexin_2_Receptor_Agonist	0.9967	−10.8	0.9955
Ponatinib	0.9964	−11.4	0.9921
DJ-V159	0.9928	−11.8	0.997
Flumethrin	0.9914	−10.7	0.9979

Considering that in some cases the high docking score may be due to dominant non-specific hydrophobic interactions, we also selected candidates with criteria of DeepBindBC>0.99, Docking<=−8.5, and DFCNN>0.998, which resulted in extra 24 compounds, as shown in Table 2. We found that many compounds are high potential binders of TIPE2 according to DeepBindBC and DFCNN. For instance, the UM-164 has a DeepBindBC score of 0.9968 and a DFCNN of 0.9994.

**TABLE 2:** The potential inhibitors of TIPE2 from the TargetMol dataset (DeepBindBC>0.99, Docking<=−8.5, and DFCNN>0.998, excluding compounds in Table 1). The table was ranked by docking score in descending order.

Name	DeepBindBC	Docking	DFCNN
ARRY380	1	−10.4	0.9982
Probucol	0.9997	−9	0.9995
Radotinib	0.9986	−10.1	0.9988
FIIN-3	0.9982	−9.8	0.9993
Rociletinib	0.9979	−10.6	0.9987
Torin_1	0.997	−9	0.9993
UM-164	0.9968	−10.9	0.9994
Fenretinide	0.9967	−9.2	0.9981
Fedratinib	0.9965	−9.7	0.9993
GNF-7	0.9964	−12.9	0.9991
DCC-2036	0.9959	−10.2	0.9995
AST_487	0.995	−10.5	0.9995
FIIN-2	0.995	−10.8	0.9994
Flumatinib	0.9947	−11	0.9994
Golvatinib	0.9942	−10.9	0.9993
CHMFL-BMX-078	0.9929	−9.4	0.9994
DDR1-IN-2	0.9925	−11.7	0.9991
K_0859	0.9922	−11.4	0.9994
A_740003	0.992	−9	0.9981
ZCL_278	0.9919	−9	0.9989
Dehydroandrographolide succinate	0.9918	−8.5	0.9993
Nilotinib	0.9916	−11.2	0.9993
TG101209	0.9915	−9.9	0.9994
Masitinib	0.9903	−10.2	0.9983

At the same time, we also obtained 22 candidates from the in-house TCM dataset, with the criteria of DeepBindBC>0.9, Docking<=−6, and DFCNN>0.9. Since the TCM ingredients are an important source of many drug leads, it would be attractive if there are some active compounds against TIPE2 by binding. Among the 22 TCM ingredients in Supplementary Table S1, kurarinone and astaxanthin have a high chance of binding with TIPE2 according to the prediction scores of DeepBindBC and DFCNN; both scores are above 0.99. Their docking scores are −9.4 and −10.8, respectively, which support their potential high-affinity binding.

### Fine Screening by Molecular Dynamics Simulation and Metadynamics Simulation-Calculated Free Energy Landscape

**FIGURE 3:**
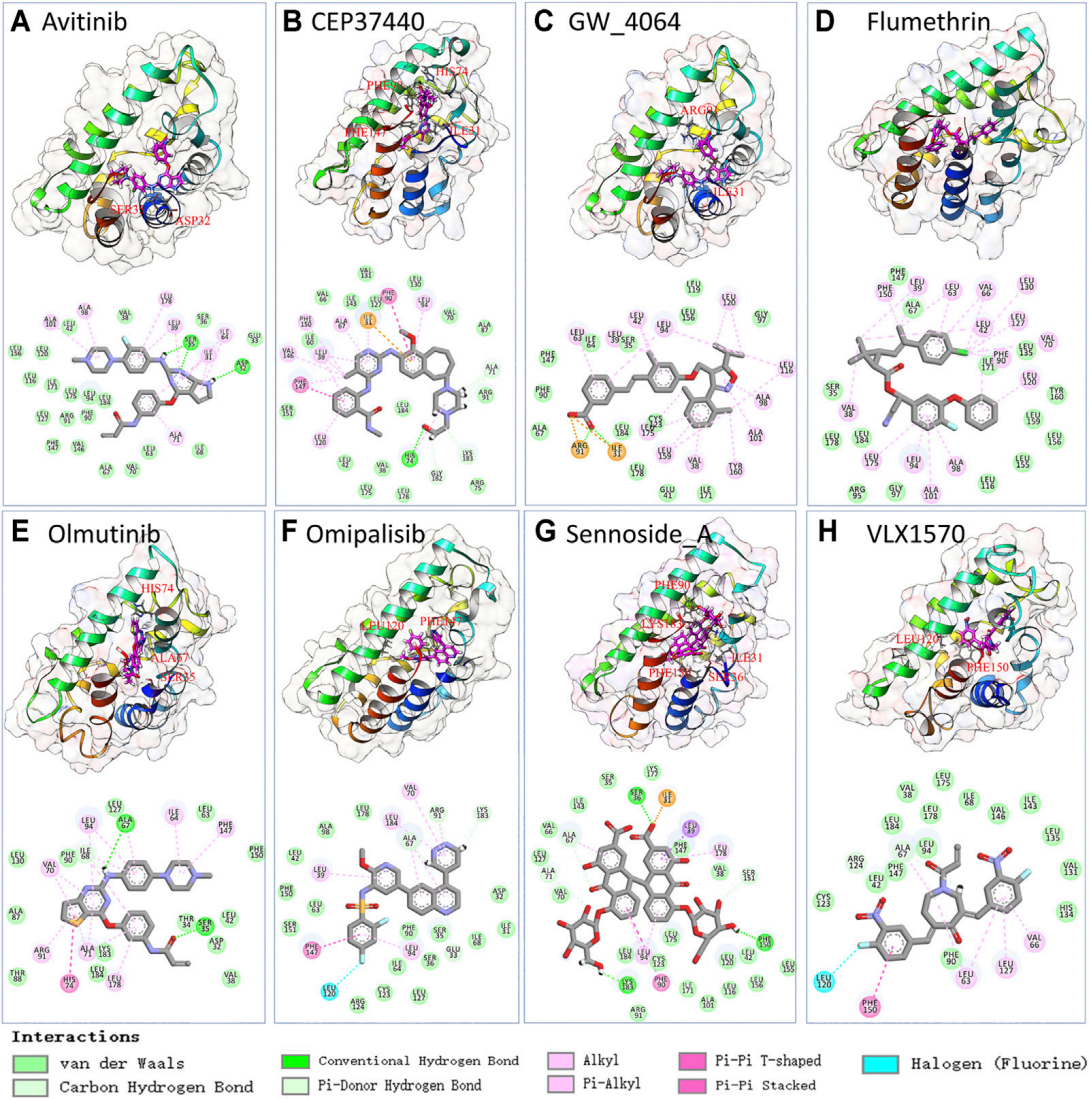
The snapshot and 2D interaction of last frame conformation from the 100 ns MD simulations trajectories of candidate list 1.

To further validate the ligand binding stability and confirm the atomistic interaction details of the above-selected candidates with the TIPE2, a 100 ns molecular dynamics simulation was carried out for each selected complex. The binding stability was mainly estimated by RMSD of the calculated ligand and protein C alpha carbons from the simulation trajectory. The top eight stable ligand RMSD from list 1 are shown in Supplementary Figure S2. The hydrogen bond numbers over the simulation time were also shown. The detailed atomic interaction of these eight complexes from the last frame of MD simulation is displayed in Figure 3. The RMSD of the top eight most stable ligands from list 2 are shown in Supplementary Figure S3A. The corresponding hydrogen bonding number with the protein along the simulation time is shown in Supplementary Figure S3B.

**FIGURE 4:**
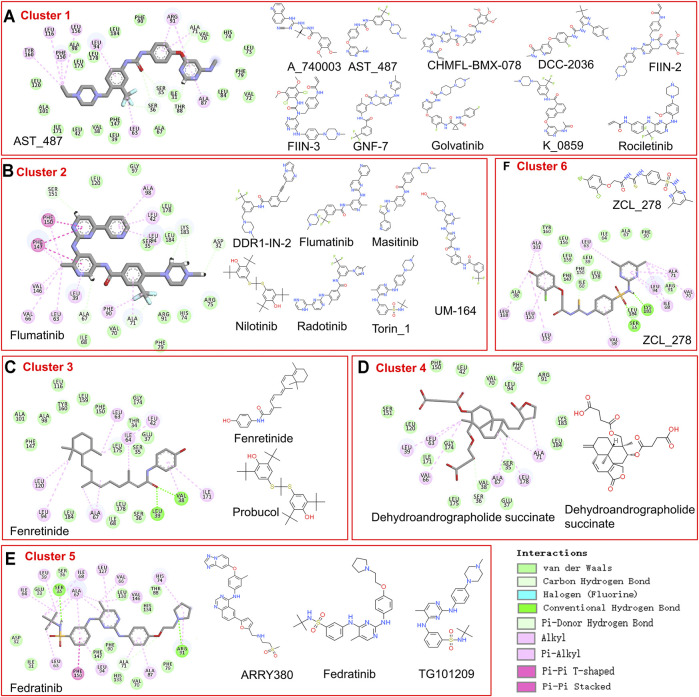
The compounds in six clusters of list 2 and the 2D interaction of each representative compound with the binding pocket.

**FIGURE 5:**
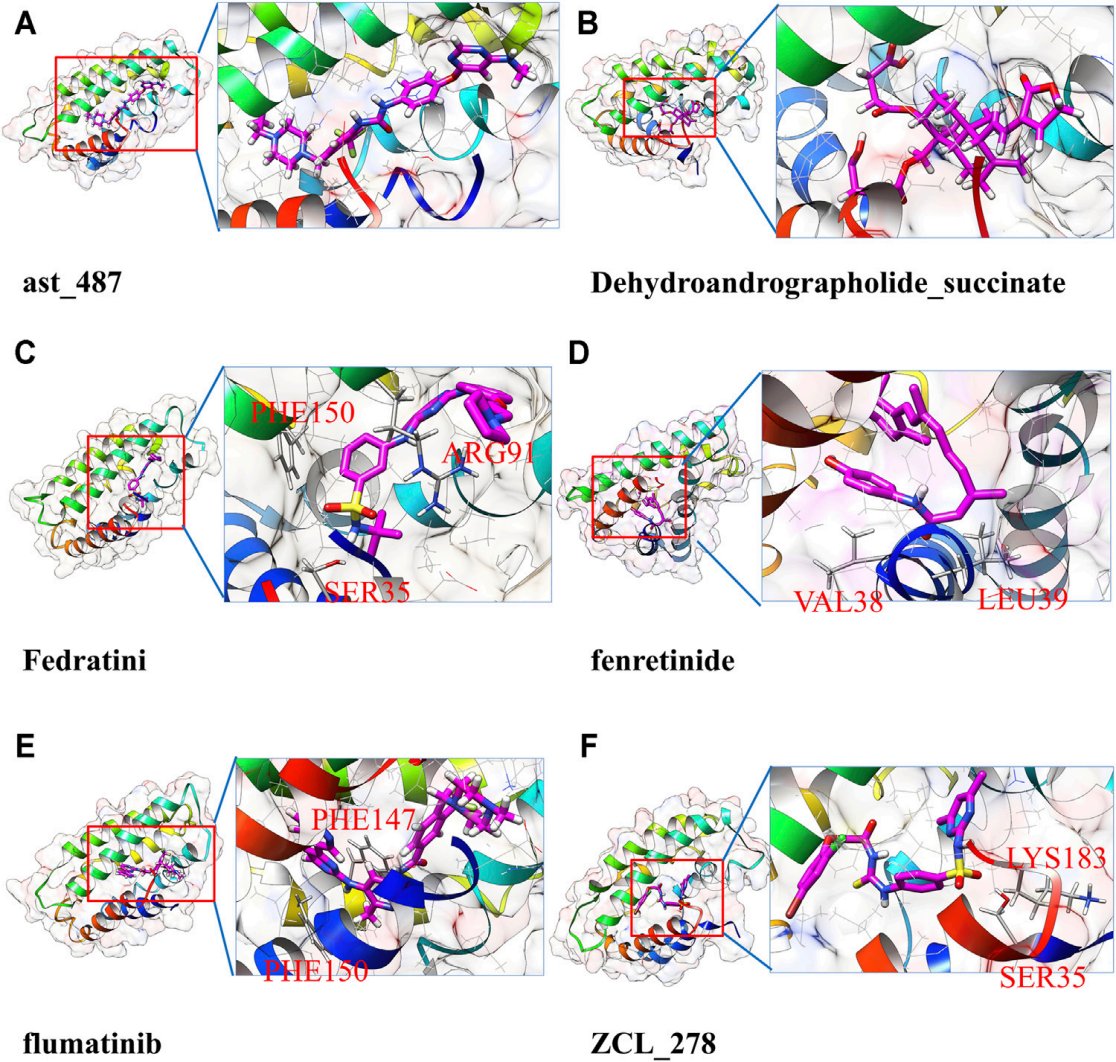
Detailed atomic interaction between TIPE2 and six representative ligands of candidate’s list 2 in the last frame of MD simulation.

As too many potentially inhibiting compounds were found in our research, we clustered them into six groups and focused on the representative compounds of each group to better present the results, as shown in Figure 4. The compounds in the same cluster show very similar structural conformation and physical-chemical properties. It was found that cluster 1 and cluster 2 contain a large number of predicted candidates, while the representative structure of these two clusters shows that hydrophobic interaction has a dominant contribution. The representative structures of clusters 3, 5, and 6 have formed hydrogen bonds with the TIPE2 according to the 2D interaction plot for the last frame of MD simulation. Furthermore, we displayed snapshots of TIPE2 and six representative ligands with detailed atomic interactions from the last frame of MD simulation in Figure 5, respectively. It can be seen that the six representatives have accommodated well with the TIPE2 pockets, which indicates that all the six representatives have a high chance of binding with the TIPE2.

**FIGURE 6:**
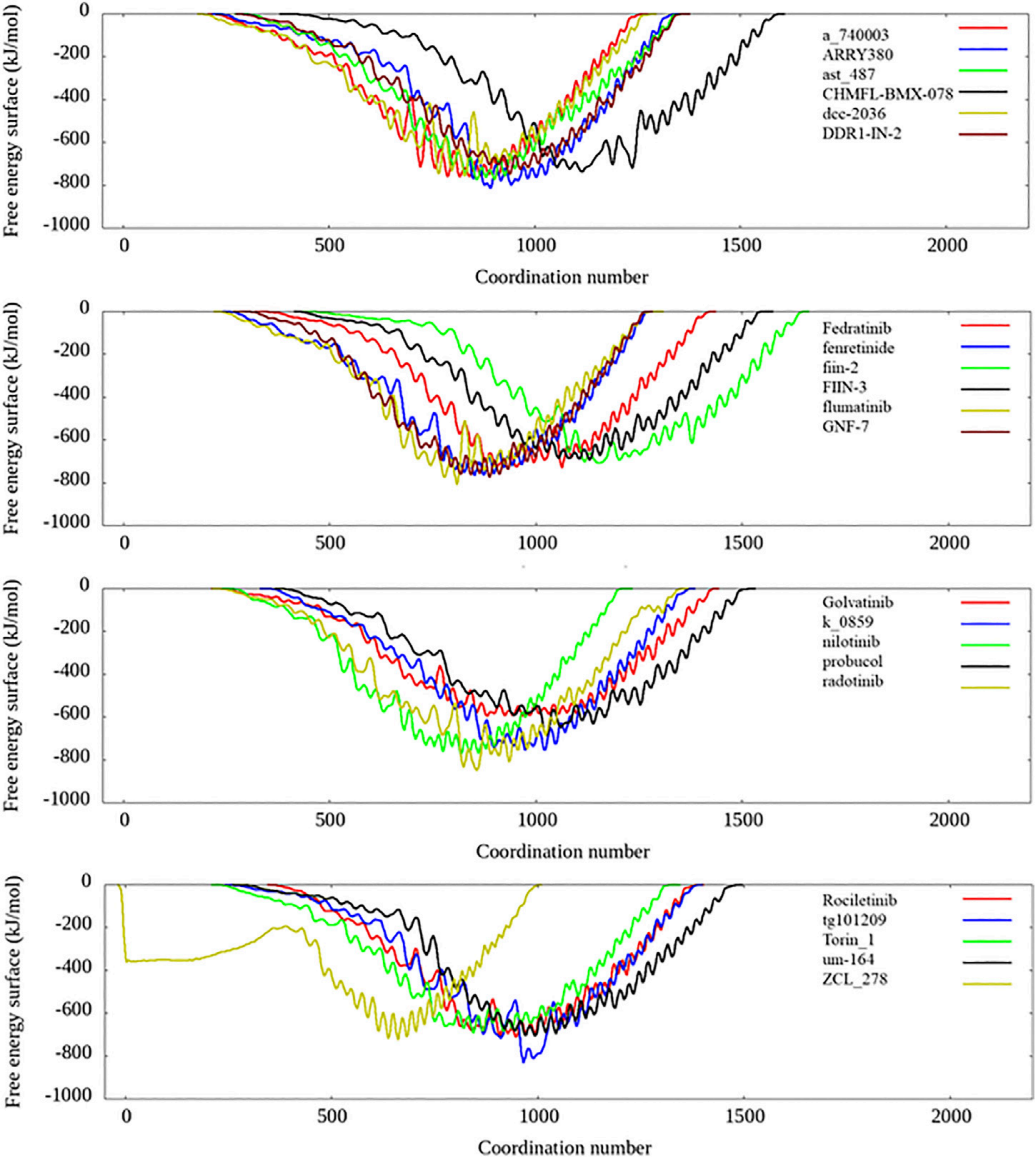
The free energy landscape of several candidates from candidate lists 1 and 2.

To more accurately estimate the binding preference of the selected ligand candidates to TIPE2, we have examined the calculated free binding free energy landscape from the metadynamics simulation. Most candidates in list 1 and list 2 were high potential binders according to the calculated binding free energy, and their binding free energy landscape was shown in Figure 6. Among the seven selected candidates from the in-house TCM dataset, four ingredients show favorable binding with the TIPE2 according to the free energy landscape from the metadynamics simulation, as shown in Supplementary Figure S4.

**FIGURE 7:**
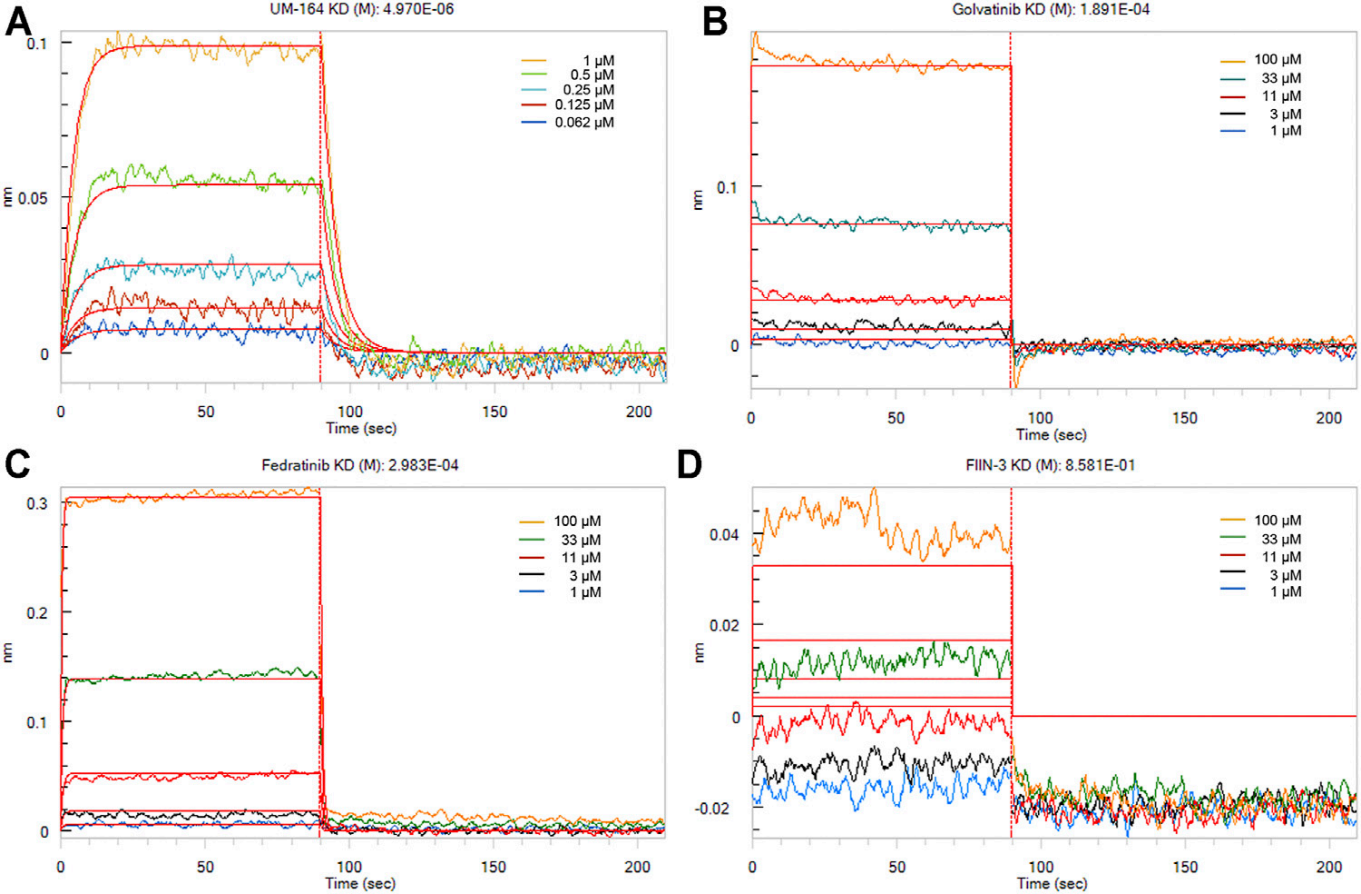
The binding affinities of small molecules with TIPE2. TIPE2 proteins were loaded onto SSA biosensors for BLI analysis and incubated with gradient concentrations of UM-164 **(A)**, Golvatinib **(B)**, Fedratinib **(C)**, and FIIN-3 **(D)**, respectively. The raw binding curves at different concentrations are shown in five kinds of color, and red curves are the best global fits to the data used to calculate the K_D_ values.

To measure the binding affinities of these small molecules, bio-layer interferometry using recombinant TIPE2 proteins was performed. The bio-layer interferometry (BLI) technique is extremely valuable and one of the most authoritative methods to estimate protein-ligand binding affinity (Zhou et al., 2018; Abdul Azeez et al., 2019; Maji et al., 2019). As shown in Figure 7, UM-164 was able to bind to the TIPE2 protein in appreciable potency, with a K_D_ value of 4.97 µM, while Fedratinib and Golvatinib were bound to TIPE2 proteins with K_D_ values of 298 and 189 μM, respectively. Nevertheless, FIIN-3 exhibited a lack of reactivity against TIPE2 protein.

### Inhibition Efficacy of UM-164

**FIGURE 8:**
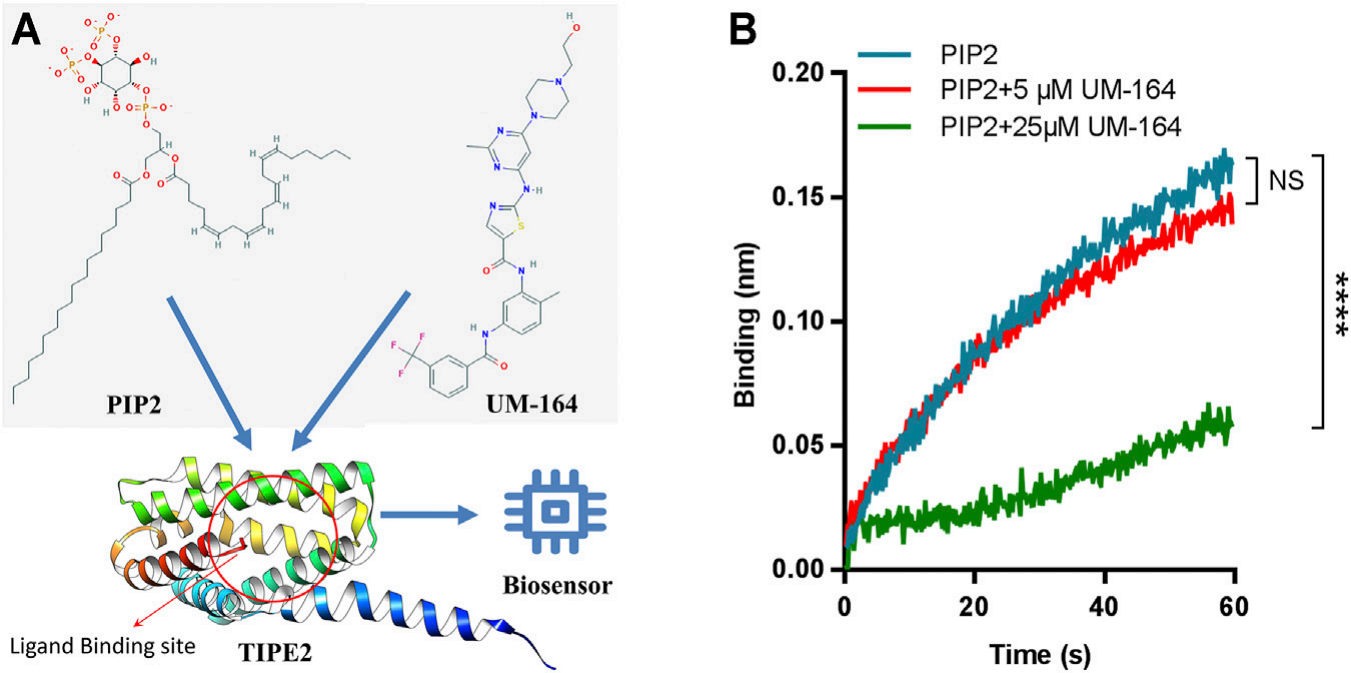
Competitive binding to TIPE2 protein between PIP2 and UM-164. **(A)** Diagram of PIP2 and UM-164 competitive binding toTIPE2. **(B)** The competitive BLI results. For BLI assays, immobilized biotinylated TIPE2 proteins were bound to PIP2 in the presence of 5 μM UM-164 (red) and 25 μM UM-164 (green) and without UM-164 (blue), respectively, and then respective binding signals of PIP2 were shown. ****: compared with the group without UM-164, *p* < 0.0001 (unpaired t-test). NS, no significance.

To assess the ability of UM-164 to inhibit the interaction between TIPE2 and PIP2, competitive binding assays were performed by BLI. Immobilized biotinylated TIPE2 proteins were saturated with PIP2 in the presence of 5 and 25 μM UM-164 or in the absence of UM-164, respectively. Compared with 5 μM UM-164 and without UM-164, when 25 μM UM-164 was added to the assay buffer, a dramatic reduction of PIP2 bound to TIPE2 protein was observed (Figure 8) possibly because UM-164 and PIP2 may recognize the same or overlapped site on TIPE2 protein, or allosteric effects, and therefore UM-164 was able to interfere with the binding of TIPE2 and PIP2. These results indicate that UM-164 is a potential inhibitor against TIPE2 function.

## Discussion

Constructing a computational-aided drug screening pipeline can greatly facilitate the process of finding drug leads. Finding effective inhibitors with limited experiments would be a challenging task. We have already performed virtual screening with a hybrid method of deep learning and molecular dynamics simulation in our previous work. Together with a previous SARS-Cov2 drug repurposing work, we further show the workable and high efficiency of the hybrid screening strategy. With the high efficiency of deep learning and relative accuracy of MD simulation, our drug screening pipeline demonstrated good performance. Deep learning and docking-based methods can greatly narrow down the candidate pool with affordable computational cost. Since the TIPE2 has a relatively small size and a large ligand binding cavity, it is possible for us to run relatively large-scale MD simulations and metadynamics simulations that can lead to a more accurate prediction.

MD simulations provide more accurate atomic details of the protein-ligand interaction, making the prediction much more explainable, and help gain insights into the mechanisms of ligand binding. Taking the MD simulation part alone, it is the first time that we carried more than 60 MD simulations for screening inhibitors against a given protein target. Thanks to the increasingly available computational resources nowadays, the MD simulation would play a much important role in future drug screening.

It is the first time that a low-nanomolar affinity binder of TIPE2 (UM-164) has been discovered, and its binding is strong enough to block the binding of native ligand PIP2, indicating that the UM-164 can inhibit TIPE2’s function. To explore which kinds of ligands prefer binding with the TIPE2, we have carefully checked the atomic interaction of candidates binding with TIPE2. We also provide a large number of TIPE2 inhibitor candidates, which can greatly promote the future discovery of new inhibitors by future experimental validation. Together, it may promote the future development of novel drugs that may cure lung cancer.

Compared to ligand-based drug screening, such as LigandScout (Wolber and Langer, 2005), our pipeline can select potential drugs with a much wider chemical space, which greatly promotes real novel drug development. As we can see from Figure 3, the potential TIPE2 binding ligands found by our method are diverse and have been divided into several categories. Compounds with quite different structures can bind into the same target, while traditional screening-based on ligand similarity cannot achieve this. Candidates with different structures provided the chance to develop diversified drugs.

## Conclusion

With the help of the hybrid drug screening pipeline, we found 69 drug candidates against TIPE2 function. We selected four candidates for final experimental tests, in which UM-164 could bind to TIPE2 with low-micromolar affinity (K_D_ = 4.97 µM). In addition, Golvalinib and Fedratinib were bound to TIPE2 with K_D_ values of 189.1 and 298.3 µM, respectively. Only FIIN-3 has a weak binding affinity with a K_D_ value of 8.581 × 10^−1^ M. This greatly expanded the available inhibitors for TIPE2, which is a potential novel drug target related to cancer and inflammation. Moreover, the high success rate of the hybrid drug screening pipeline indicates a huge potential for the implementation of similar methods in the drug discovery of other targets. The active compounds we found and their possible derivatives have the potential to facilitate drug development for TIPE2. The detailed interaction between those inhibitors and TIPE2 also provides insight into understanding the binding mechanism and drug design and refinement.

## Data Availability

The original contributions presented in the study are included in the article/Supplementary Material; further inquiries can be directed to the corresponding authors.
