# Supplementary material for: An Integrated Deep Learning and Molecular Dynamics Simulation-Based Screening Pipeline Identifies Inhibitors of a New Cancer Drug Target TIPE2
Source: Front Pharmacol. 2021 Nov 23;12:772296. doi: 10.3389/fphar.2021.772296 (PMC8650684; doi:10.3389/fphar.2021.772296)
Supplement: Supplementary file 1 [file DataSheet1.docx]

**Supplementary material section 1:**

**Detailed procedure of force field-based screening**

MD simulation was carried out by Gromacs with AMBER-99SB force field (B Hess, Kutzner, & Spoel, 2008; Hornak & Simmerling, 2003). The topology of the ligand and the partial charges of the ligand were generated by ACPYPE (Sousa Da Silva & Vranken, 2012), which relies on Antechamber (Wang, Wang, Kollman, & Case, 2006). Firstly, we created a dodecahedron box and put the target-ligand complex at the center. A minimum distance from the protein to the box edge was set to 1 nm. We filled the dodecahedron box with TIP3P water molecules (Jorgensen, Chandrasekhar, Madura, Impey, & Klein, 1983), the counterions were added to neutralize the total charge using the Gromacs program tool (Van Der Spoel et al., 2005). The long-range electrostatic interactions under the periodic boundary conditions were calculated with the Particle Mesh Ewald approach (Darden, York, & Pedersen, 1993). A cutoff of 14 Å was used for van der Waals non-bonded interactions. Covalent bonds involving hydrogen atoms were constrained by applying the LINCS algorithm (Berk Hess, Bekker, Berendsen, & Fraaije, 1997).

We performed the energy minimization steps with a step-size of 0.001ns, 100 ps simulation with isothermal-isovolumetric ensemble (NVT), and 10ns simulation with isothermal-isobaric ensemble (NPT) for water equilibrium. After that, a 100ns NPT production run (step size 2 fs) was carried out. The Parrinello-Rahmanbarostat and the modified Berendsen thermostat were used for simulation with a fixed temperature of 308 K and a pressure of 1 atm. RMSD and hydrogen bond number of the trajectory were calculated using Gromacs tools.

The simulation was continued using the metadynamics approach for exploring the free energy landscape. The interface coordination number of atoms of protein ligand complex was used as a collective variable (CV). The protein-ligand interface coordination number of atoms correlates with the numbers of atom contact, and a larger coordination number usually indicates protein-ligand is in a binding state.

The coordination number *C* is defined as follows by Plumed:

$C=\sum_{i\in A}\sum_{j\in B}S_{ij}$（1）and

$S_{ij}=\frac{1-\left(\frac{r_{ij}-d_{0}}{r_{0}}\right)^{n}}{1-\left(\frac{r_{ij}-d_{0}}{r_{0}}\right)^{m}}$（2）

In the simulation, *n* was 8, *m* were 12, $d_{0}$ was 0 nm and $r_{0}$ was 0.25 nm. $d_{0}$ is a parameter of the switching function. $r_{ij}$ is the distance between atom *i* and atom *j*. The degrees of contact between two groups of atoms can be estimated by the function (Tribello, Bonomi, Branduardi, Camilloni, & Bussi, 2014). Metadynamics simulation for each protein-ligand system was performed for 100 ns (except protein-Azithromycin, which was extended to 300ns in order to reach the 0 Coordinate Number and achieve convergences). During the metadynamics simulation, Gaussian values were deposited every 1 ps with a height of 0.3 kJ/mol. The widths of the Gaussians were 5 for the coordination number. The free energy landscapes of the metadynamics simulations along the CV were generated by the Plumed program and plotted using Gnuplot (Williams et al., 2012).


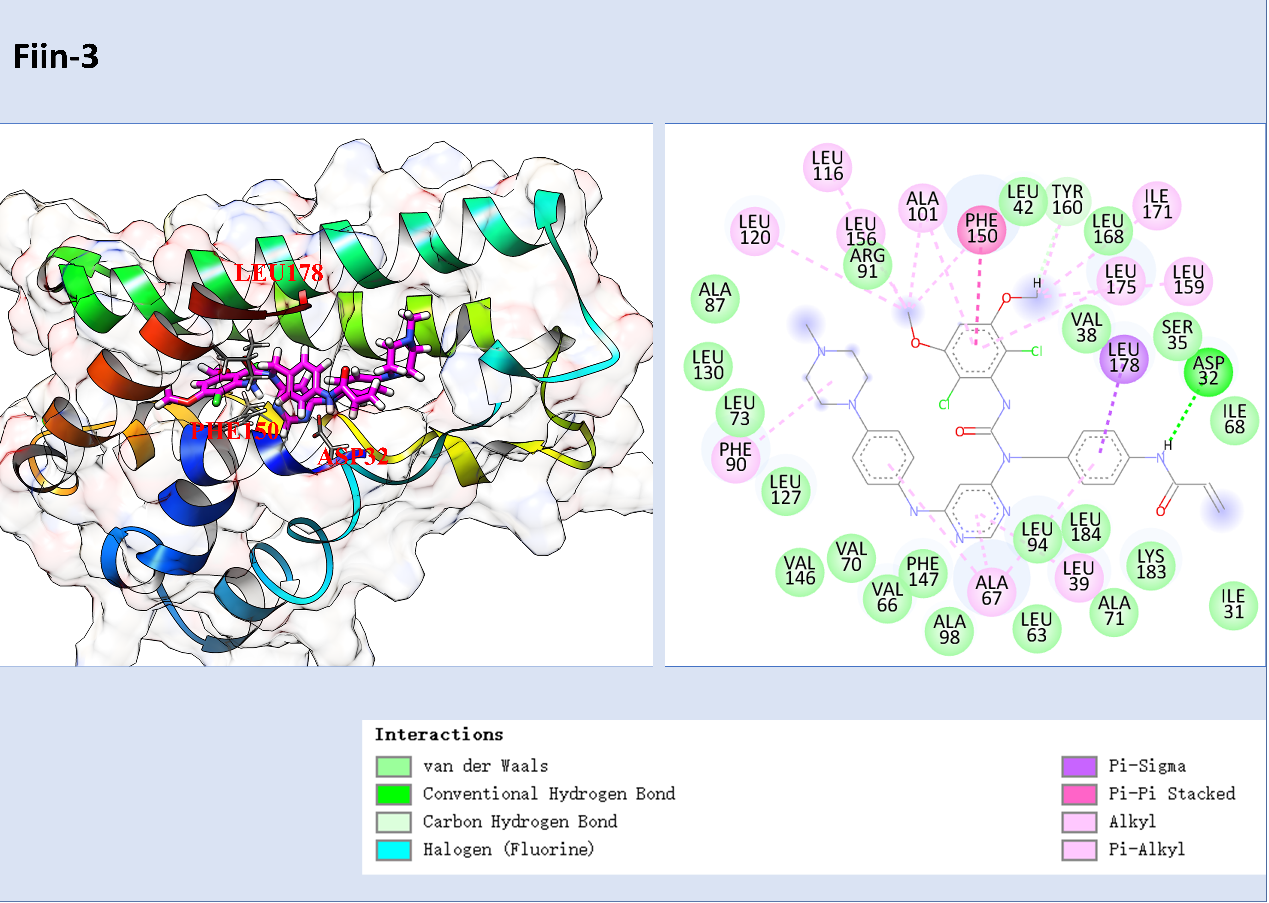


Figure S1. Display the predicted atomic interaction details of Fiin-3 with TIPE2.


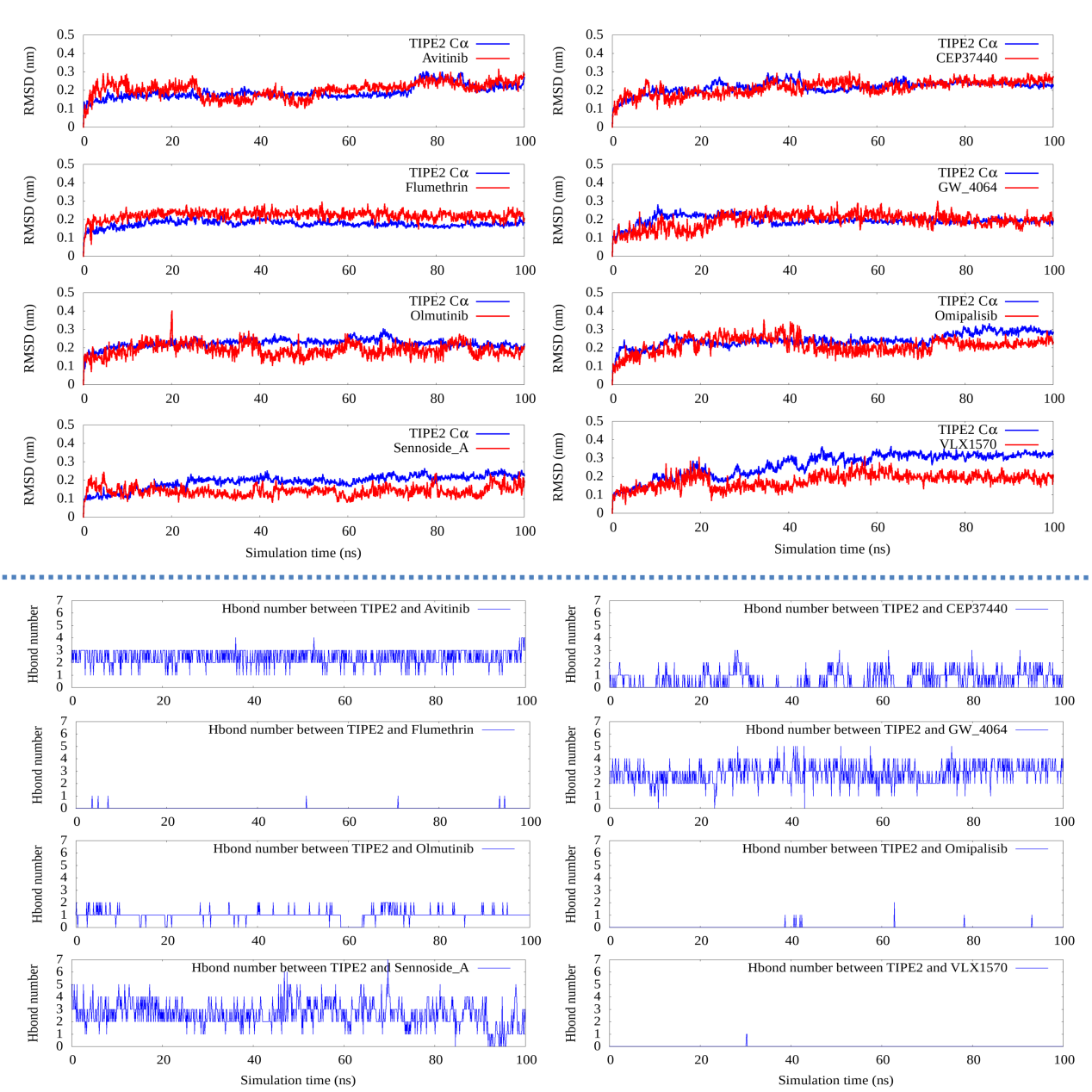


Figure S2. display some cases of candidate list 1 with relatively low RMSD fluctuation and their corresponding hydrogen bind numbers between ligand and protein from MD trajectory.


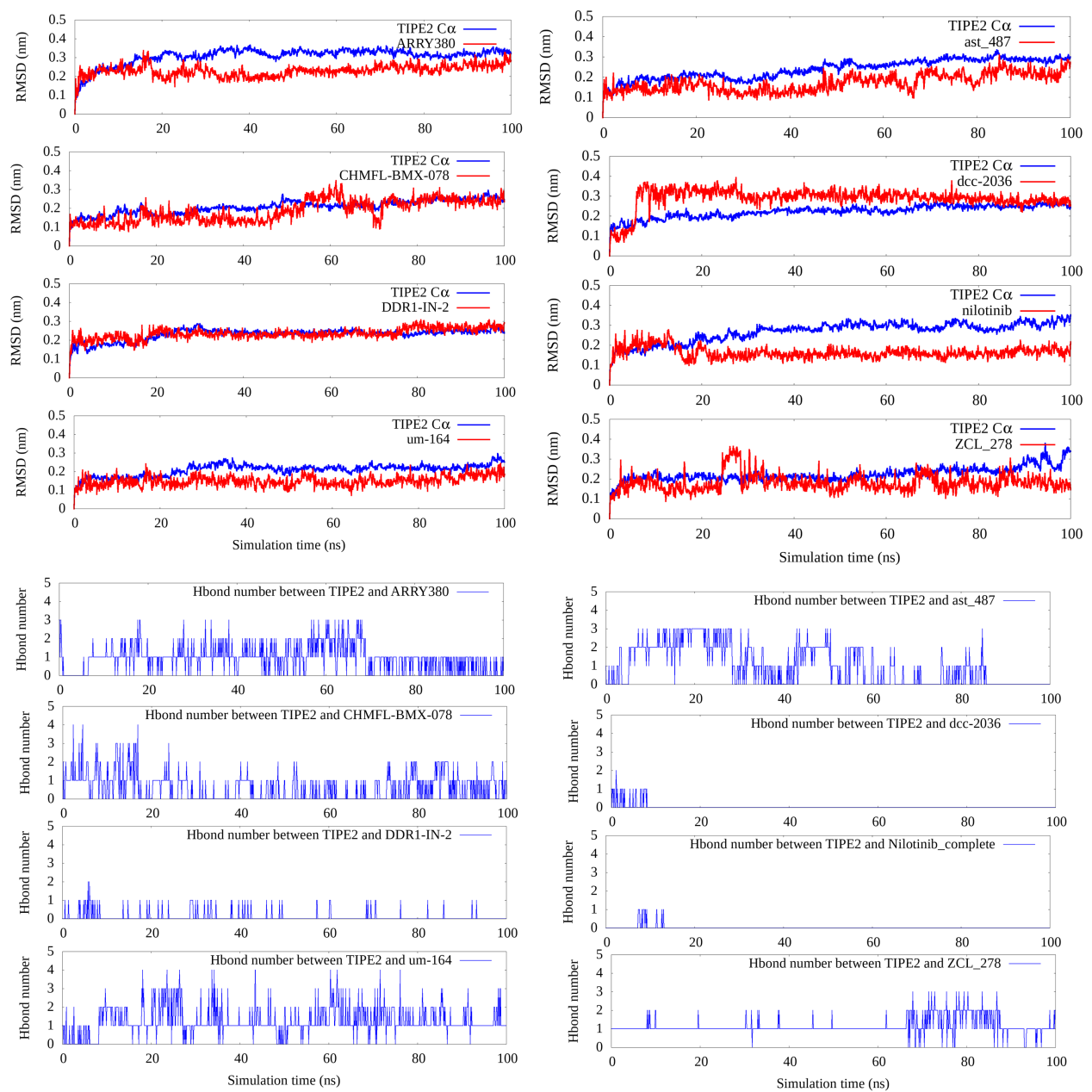


Figure S3. shows some cases with relatively low RMSD fluctuation and their corresponding hydrogen bind numbers between ligand and protein from MD trajectory.


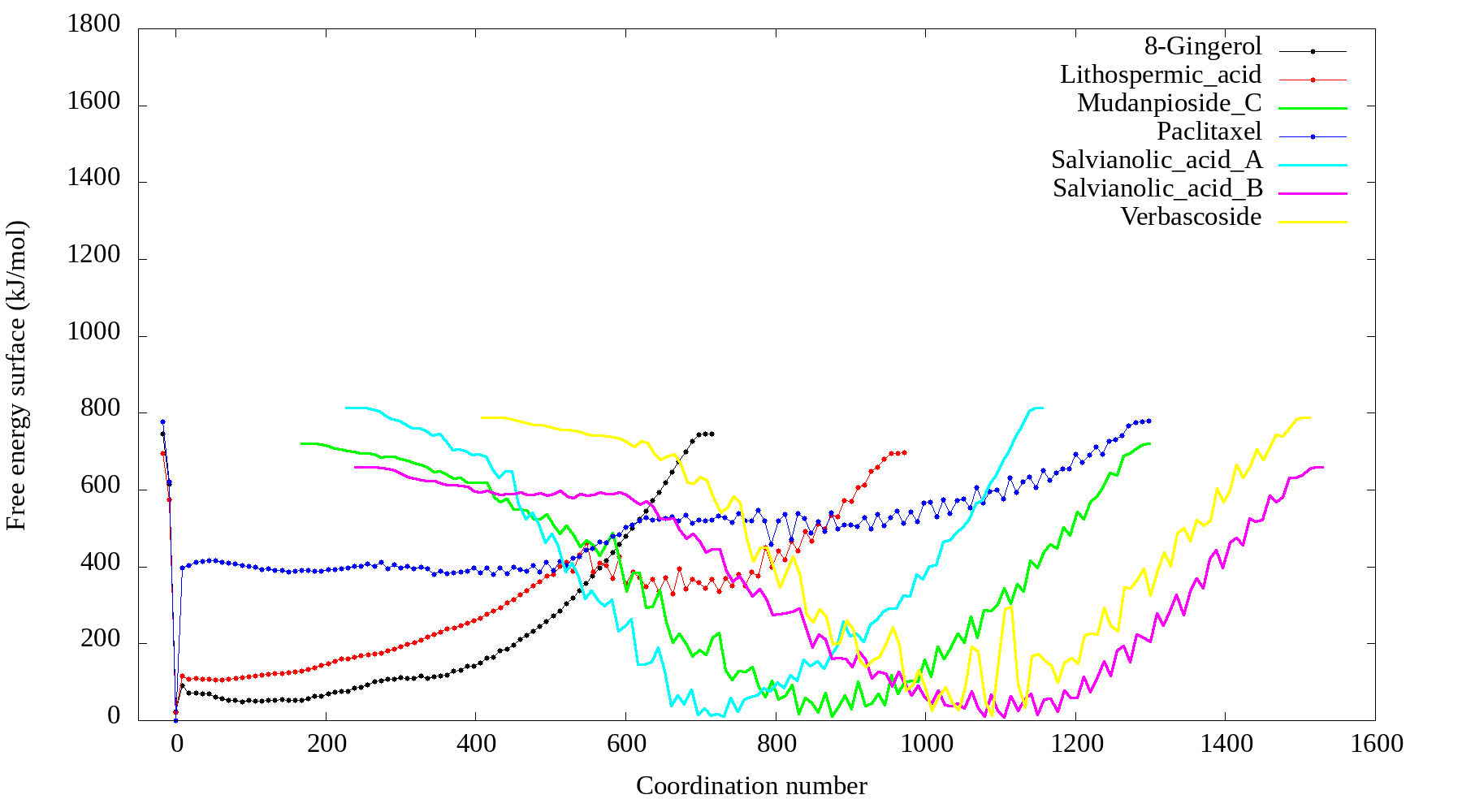


Figure S4. The free energy landscape of several candidates from the in-house TCM dataset.

Table S1. The potential inhibitors of TIPE2 from the in-house TCM dataset (DeepBindBC>0.9, Docking<=-6, DFCNN>0.9).

| Name | DeepBindBC | Docking | DFCNN |
| --- | --- | --- | --- |
| praeruptorin_B | 0.9997 | -9.3 | 0.9428 |
| Kurarinone | 0.9983 | -9.4 | 0.9977 |
| Astaxanthin | 0.998 | -10.8 | 0.9998 |
| Praeruptorin_D | 0.9966 | -9.5 | 0.9428 |
| Docetaxel | 0.9965 | -7.8 | 0.9812 |
| Salvianolic_acid_B | 0.9958 | -8.7 | 0.976 |
| Salvianolicacid_C | 0.9947 | -9.8 | 0.92 |
| Paclitaxel | 0.9942 | -8.1 | 0.9777 |
| Lithospermic_acid | 0.992 | -8.8 | 0.9745 |
| Cynarin | 0.9852 | -8.6 | 0.9977 |
| Isochlorogenic_acid_B | 0.9849 | -9 | 0.9969 |
| Tenacissoside_I | 0.9848 | -9.7 | 0.9009 |
| 6'''-Feruloylspinosin | 0.9722 | -8.6 | 0.9686 |
| Verbascoside | 0.9652 | -8.9 | 0.9267 |
| Tenacissoside_H | 0.9631 | -7.9 | 0.9065 |
| Demethylzeylasteral | 0.9626 | -7 | 0.9955 |
| Gomisin_G | 0.9615 | -8.7 | 0.9771 |
| Eriocitrin | 0.9585 | -8.4 | 0.9097 |
| Mudanpioside_C | 0.9545 | -8.8 | 0.9811 |
| Yejunualactone | 0.9506 | -6.9 | 0.9657 |
| Salvianolic_acid_A | 0.9498 | -9.2 | 0.9873 |
| 8-Gingerol | 0.9456 | -8.1 | 0.9588 |
